# Practical Methodological Reform Needs Good Theory

**DOI:** 10.1177/1745691620977471

**Published:** 2021-01-29

**Authors:** Will M. Gervais

**Affiliations:** Centre for Culture and Evolution, Department of Psychology, Brunel University London

**Keywords:** metascience, methods reform, philosophy of science, cultural evolution, modeling, norms, diversity and inclusion, theory

## Abstract

In the face of unreplicable results, statistical anomalies, and outright fraud, introspection and changes in the psychological sciences have taken root. Vibrant reform and metascience movements have emerged. These are exciting developments and may point toward practical improvements in the future. Yet there is nothing so practical as good theory. This article outlines aspects of reform and metascience in psychology that are ripe for an injection of theory, including a lot of excellent and overlooked theoretical work from different disciplines. I review established frameworks that model the process of scientific discovery, the types of scientific networks that we ought to aspire to, and the processes by which problematic norms and institutions might evolve, focusing especially on modeling from the philosophy of science and cultural evolution. We have unwittingly evolved a toxic scientific ecosystem; existing interdisciplinary theory may help us intelligently design a better one.

There’s nothing so practical as a good theory.

— Kurt Lewin (1943, p. 118)

If you want science to be unquestionable, it isn’t really science that you want.

— Iris van Rooij (2020)

Many sciences are a-changing. Spurred by a flurry of unsuccessful replications of prominent work, exposure of scientific fraud and negligence bordering on fraud, and publication of highly implausible research, psychological science has been at the heart of a movement described variously as the “Open Science movement,” the “credibility revolution,” or the “methodological reform movement” (Spellman, 2015). This movement has been typified by an eager, roll-up-your-sleeves, bottom-up push for stronger methodological practices in psychology. Core statistical practices, scientific philosophies, and publishing norms have been challenged, reframed, and rebuilt on the fly. Promising innovations and tweaks have sporadically emerged, and more emerge daily. This groundswell embodies boundless enthusiasm but has faced occasional strife. The reform and metascience movement faces complex challenges amid fields-wide disputes over the (a) severity of scientific shortcomings, (b) ultimate reform goals, and (c) optimal mechanisms for achieving those goals. Without theoretical maps for navigating this morass, metascience and methodological reform movements—despite noble intentions—risk needlessly reinventing other fields’ time-tested wheels or, worse, reproducing the problematic norms, institutions, and incentives of status quo science.

With this in mind, I offer some observations and recommended resources, often from fields outside of mainstream psychology, that may have been somewhat overlooked in the eager embrace of methodological reform by psychologists. Incorporating these theoretical perspectives may hasten methodological progress and save all of us reformers some time, given that theory will let us focus on the most productive avenues and also pretest interventions using established mental prostheses such as formal models and simulations. This piece is intended to be more provocative than comprehensive, as fodder for continued improvements in psychological science. I hope it is read in the spirit it was written: I believe that a movement that has emerged from critical reflection on psychological science should be open to critical self-reflection on its own workings and open to wisdom and critiques from other fields that may have important theoretical insights. Not only do we not have all the answers, but we also might not even know the appropriate questions to ask.

I begin by briefly surveying the state, such as it is, of metascientific and methodological reform theory on the basis of prominent and recent publications. Next, I discuss two potential avenues for injecting theory into methodological reform. I cover recent advances in theoretical approaches to scientific aspirations—what does theory say about how to structure a more efficient and equitable science? Finally, I offer theoretical insights from the cultural-evolution literature on how cultures, norms, and institutions change; it seems plausible that a theoretical knowledge of mechanisms underlying norm and culture change in general might be useful for specific targeted changes to scientific norms and culture.

## The State of Theory in Psychology Metascience

Despite this large mass of data . . . I found myself puzzled as to what a rational mind ought to conclude about the state of the evidence.

— Paul Meehl (1990, p. 195)

An overarching stated goal of metascience and reform efforts in psychology is to improve the replicability and robustness of psychological science. This makes large-scale replication efforts and replication work a convenient place to examine the strength of theory in the reform movement. Given that these efforts have been in full swing for at least a decade or so by now, it is possible to take a preliminary assay of reform and metascience work in psychology to consider its theoretical grounding. So, how much of psychology research is replicable?

### What is replicable?

A 2015 *Science* article, grandiosely entitled “Estimating the Reproducibility of Psychological Science,” sought to replicate 100 studies and (depending on which definition is adopted), and around 2 in 5 were successfully replicated. Does this mean 40% of psychology is replicable?

Not so fast.

This original *Science* article offered no fewer than five potential criteria for assessing whether a given study has successfully been replicated:

1. The replication is statistically significant (*p* < .05) in the same direction as the original.
2. The effect sizes are comparable.
3. There is a significant result when original and replication effects are meta-analytically combined.
4. *P*(UpperCI_replication_ > Point_original_ > LowerCI_replication_).
5. The team members’ subjective appraisal is positive.

This taxonomy of replication has been supplemented by other suggestions, including (but presumably not limited to):

6. *P*(UpperCI_original_> Point_replication_ > LowerCI_original_) (Gilbert et al., 2016).
7. The original studies have power to detect replication efforts (Simonsohn, 2015).
8. Bayes factors yield strong evidence in support of an alternative hypothesis (Etz & Vandekerckhove, 2016).
9. Evidence is updated via replication Bayes factors (Ly et al., 2019).
10. The existence of presumably positive results from any “study for which any outcome would be considered diagnostic evidence about a claim from prior research” (Nosek & Errington, 2020, para. 6).
11. The degree to which the original and replication effect sizes significantly differ from each other (Srivastava, 2012).

Definitional quibbles aside, the *Open Science Collaboration* article cannot even in principle achieve its stated goal of estimating the reproducibility of psychological science given its sampling procedure. This collaboration assembled a semirandom set of *studies available for potential replication*. The initial set consisted of studies from a given year published in *Journal of Personality and Social Psychology*, a flagship social/personality journal; *JEP: Learning Memory and Cognition*, a flagship cognitive journal; and *Psychological Science*, a flagship general psychology journal. Entire branches of psychology—developmental, neuroscience, clinical, comparative, evolutionary, cross-cultural, etc.—were largely invisible to this project. From this pool, volunteer teams could register to tackle a given study. When the target of 100 studies was not reached, the core replication team reached out individually to solicit researchers to conduct specific replication studies. Although the solicitation/volunteer status of each replication was not coded, Nosek commented that half might have resulted from direct contact and study suggestion (Nosek, 2016). The resulting project was an impressive organizational effort, provides much valuable information, and likely spurred efforts to bolster methods, but it clearly cannot make general pronouncements about the replicability of psychological science as a whole.

Other efforts have taken different study-sampling approaches, either recruiting multiple labs to replicate a single protocol (the Registered Replication Report format), or many teams replicating a dozen or more easy-to-administer quick online or laboratory tasks (the Many Labs format). It is unclear, however, exactly how candidate studies were chosen for these projects. They appear to stem from easily run tasks, combined with high-visibility findings, potentially producing an overrepresentation of the literature of flashy or counterintuitive findings from what has come to be known as *social priming.* Combining all efforts, it seems like perhaps half of the attempted replication projects have yielded evidence of replicable results. Though tempting, it is not possible to directly generalize this to the broader literature, for reasons well trodden in any methodological text with a section on sampling. Beyond the technicalities of the precise rate of replicability in psychology, there remains the much thornier question of what any given obtained replicability rate value would mean, or what it should be.

Although efforts to estimate the overall replicability of psychological science remain elusive, are we at least probing the literature in a theoretically grounded manner? Prominent replication projects often target studies somewhat subjectively, on the basis of personal intuition or publication prestige. Such projects are invaluable, but unaided intuition may not optimally guide metascientific forays any more than it optimally guides researchers in primary topical research. For all research, theory is a useful mental prosthesis in selecting projects (Muthukrishna & Henrich, 2019). Only in recent years have researchers begun to outline a priori criteria for selecting replication targets. One, for example (Field et al., 2019), seeks to optimize replication value by focusing on a few key theoretical, statistical, and feasibility desiderata. This approach and others like it may prove to be useful; they may fail utterly. What is truly remarkable is that after about a decade of intense focus on replication, only recently have articles about principled ways to choose replications begun to emerge. As an anonymous reviewer of the initial submission of the manuscript for this article noted, “the reform movement in psychology has made progress only by virtue of the fact that irreproducibility is so prevalent that any researcher can stumble upon it in any meandering walk through the field. But a more systematic, principled approach is now warranted.”^1^

A generous reader at this point might be wondering, “Is it really that big a deal if replication efforts are not grounded in theory, or not dispersed optimally across the literature? Do we ask this of original research? And is the resulting inefficiency problematic? It’s the replicator’s time, after all.”

Two responses:

1. Fair enough. I do not disagree with any of that.
2. In some cases, suboptimal replication work—untethered from relevant and available theory—risks harming scientific progress.

### Metascience without theory risks harm: one example

Beyond providing guidance in the selection of replication projects, theory is absolutely essential when designing metascience projects that purport to address significant theoretical claims. One example here is Many Labs 2 (Klein et al., 2018), which sought to determine whether sample source was a moderator of experimental effects. It provided a nice test of sampling variability for an idiosyncratically selected subset of effects and perhaps surprisingly revealed that—at least for the tasks chosen—replication results were fairly consistent across volunteer sites. This would represent a blow to “hidden moderator” arguments that might dismiss a failed replication study from Topeka, Kansas, for an initial study that took place in Toledo, Ohio, for example.

However, Many Labs 2 made far more provocative claims on the basis of exploratory analyses of what they dubbed “WEIRDness,” a measure of which did not significantly moderate rates of successful replication. They adopted the WEIRDness term from the famous WEIRD-people article (Henrich et al., 2010), which coined the silly acronym WEIRD to encapsulate the ways in which typical psychology (and other social science) samples diverge from human typicality: Our samples tend to be nonrepresentatively Western, educated, industrialized, rich, and democratic. As Dan Sznycer pithily puts it, “WEIRD was penned as a memorable thing. A reminder to think about human diversity. Like RSVP. Not a concept or an explanation. A good idea, since you’ll miss a lot if you study only undergrads” (Sznycer, 2020). Nonetheless, Many Labs 2 treated the acronym as a construct, smooshed together archival indices of the letters that make up WEIRD, performed a mean split to classify samples as WEIRD and less WEIRD, and used this artificial dichotomy as a potential moderator, yielding nonsignificant results. The Many Labs 2 team featured this analysis in the abstract of the article and discussed it prominently when promoting the article’s publication. Senior author Brian Nosek called the results he obtained from the WEIRDness analysis “particularly stunning” (Nosek, 2018). Perhaps as a result, Many Labs 2’s broadest and weirdest claims are already being discussed in the press and on social media to the effect that the WEIRD-people problem is overblown.^2^

**Fig. 1.**
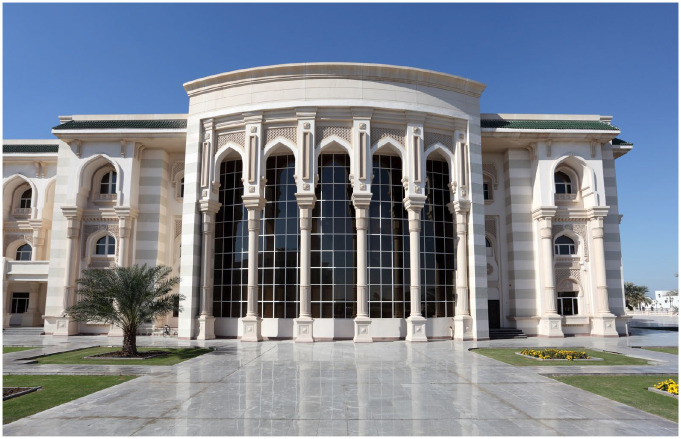
The University of Sharjah.

This dismissal of sample diversity in psychology on the basis of Many Labs 2 is unfounded. Little justification is given for the various theoretical, methodological, and statistical choices made in Many Labs 2, and the WEIRDness measure fails to deliver even face validity. English-speaking students hailing from 94 countries to attend the gold-leaf-pillared University of Sharjah (Fig. 1) were nonsensically scored as nonrich and low in education. Chile was coded in the same category as Germany and Sweden, but categorically different from near-neighbors Costa Rica and Uruguay. South Africa was coded as the same as China and India but categorically distinct from from Australia and New Zealand. Something is amiss here.

One could forgive a reader new to the social sciences—one who might be naive to the ways in which theory-driven approaches to culture tend to be able to specify (a) when cultural heterogeneity is expected (e.g., Gervais et al., 2017; Henrich et al., 2006; Kitayama & Cohen, 2010; Kline et al., 2018; Legare et al., 2012; McNamara et al., 2019; Purzycki et al., 2016; Smaldino, Lukaszewski, et al., 2019; Willard & Cingl, 2017) and (b) when homogeneity might instead be more likely (e.g., Apicella et al., 2012; Sznycer et al., 2017)—for concluding from Many Labs 2 that psychology results are generally robust across cultures.

This naive reader may understandably come to think that Many Labs 2 dealt a blow to the WEIRD-people problem—a problem that, alas, Many Labs 2 did not even tangibly address. After all, that is how it was promoted.

The seductive misinterpretation of Many Labs 2 is potentially harmful. Without theory, the interpretation “does replication success vary by location?” might seem to make sense. Without a theoretical lens through which to view culture, people may mistake an acronym (WEIRD) for a hypothesis in need of metascientific testing, and fallaciously disregard the *dramatic* lack of diversity in our science. In the current psychological science landscape, samples are overwhelmingly nonrepresentative of our species and many papers *do not even bother to identify or justify the nationality of their samples* (Cheon et al., 2020; Rad et al., 2018)—a practice now recommended but not required at our flagship journal (Bauer, 2020), a step that alas is progress. Underrepresented samples are tough to gather and then largely overlooked (Gaither, 2019) or shuttled to “specialty” niche journals (Gaither, 2020; Saab et al., 2020). Against this backdrop, there are genuine risks inherent to metascientific projects that might easily be taken—given how they are directly presented and promoted—to mean that people are essentially interchangeable and sampling diversity and inclusion are redundant at best. This threatens to further compound the WEIRD-people problem, which after all is not a mere sampling issue—it reflects and reinforces deep inequities in our field (Saab et al., 2020), further disincentivizing work on all but the most convenient of convenience samples and further distorting our science’s representation of human nature.

This section is not included to malign an exploratory analysis from one publication. We all have theoretical gaffes, and they are only to be expected in an emerging discipline such as psychology metascience. Instead, this section is included as a cautionary reminder of intellectual humility: Our zeal for metascience may be exposed as overeager when we do not appreciate existing work in relevant domains or are unwilling to engage with it. Just as Many Labs 2’s foibles concerning the nonconstruct of WEIRDness may have been averted by consultation with theory on culture, so too may theory from disciplines such as philosophy of science, philosophy of biology, and cultural evolution inform both the goals and practices of methodological reform in psychology. Scholars in these subfields have been diligently working—often for decades—to answer many of the very questions that psychology metascientists are now finding themselves asking: How do we balance key scientific desiderata? What types of structures promote or impede scientific progress? How can cultures, norms, and incentives be changed? The remainder of this article moves from discussion of psychology metascience directly to relevant work from other disciplines that have been modeling these processes for years.

### Interlude: on the Use of Models

Building a model of a thing is a wonderful way to study it . . . a model of a world. A particular world, or a possible world, or a terrible world, even.

— Kelly Sue DeConnick (2020)

Numbers are simple, obedient things, as long as you understand the rules they live by. Words are trickier. They twist and bite and require too much attention.

— Seanan McGuire (2019, p. 10)

Fight for the things that you care about, but do it in a way that will lead others to join you.

— Ruth Bader Ginsburg (Vagianos, 2015, para. 3)

Although the current state of enthusiasm in psychology methodological reform and metascience is excellent, the current state of its theory lags behind. Theory from adjacent fields that seems highly relevant to reformers (e.g., Devezer et al., 2019; O’Connor & Weatherall, 2020; Zollman, 2007) remains largely ignored or absent in discussions of psychology metascience. As a result, the energy of reformers may end up being spent in ways that are inefficient and may even be leading to harmful-but-seductive misinterpretations of the work.

To avoid the trap of using reform initiatives to create a new science that mirrors problematic aspects of the status quo in predictable ways, it is worth stepping back and considering available theoretical tools—including some from outside psychology—to ask what an ideal (or at least improved) science might look like and to then consider the processes by which cultures change in general to help chart a path from our current toxic science to whatever scientific utopia we decide we want.

The rest of the article consists of three primary sections. First, I introduce some rudimentary basics of how modeling might help answer metascientific questions using a toy model of how message framing in scientific discourse might affect uptake of scientific reforms. Second, I consider results from a wide variety of models to explore what types of sciences we might want to aspire to. Finally, I introduce a set of models from the cultural evolution literature that may be relevant for helping us intelligently design a better science.

Models have emerged as useful tools both in the philosophy of science and within cultural evolution. These models typically include transparently stated but probably unrealistic assumptions about a toy world. Theorists can construct these toy worlds for a number of reasons. First, they can explore the parameters that could in principle yield given outcomes to learn more about the kinds of interventions that can or cannot fix them. For example, O’Connor (2019b) evaluated and discussed a series of models about the processes that can generate unfairness and inequity across racial or gendered lines. She found that inequality rapidly emerges given some very sparse assumptions, without needing fancy cognitive processes such as implicit bias or stereotype threat. These models do not show, for example, that implicit bias or stereotype threat are unimportant, but they do suggest that interventions aimed solely at them will likely not solve deeper problems that generate inequities to begin with. Dropping $25,000 or more to book an expert speaker on implicit bias might feel good for an organization but not address more important structural issues. Likewise, Smaldino and McElreath (2016) modeled how incentives for productivity could lead to shoddy science; their models do not explicitly require things such as fraud, intentional gaming of the system, or nefarious intent by cynical agents. Instead, poor outcomes naturally evolve in certain scientific ecologies, given prevailing incentives. It follows from this that simply changing some practices—replicating more and more studies, for example—is just not going to ameliorate the problems (Smaldino, 2019; Stewart & Plotkin, 2020).

Models are stupid, yet useful (Smaldino, 2017). They are mental prostheses that let us check our intuitions against simplified universes. They force us to make crucial assumptions explicit so they can be openly evaluated for plausibility (Guest & Martin, 2021). They can let us specify potentially necessary and sufficient conditions to generate specific (often terrible) states of the world (O’Connor, 2019b; O’Connor & Weatherall, 2018; Smaldino, 2017), suggesting interventions that might be more or less likely to succeed. They can more starkly reveal the trade-offs that stakeholders must consider. They can illuminate otherwise unseen consequences of given actions or inactions. They can serve as yet more tools in the toolbox of aspiring metascientists. Hopefully, they can be used as aids as metascientists consider both how science ought to work and how to evolve our science in a better directions. But how exactly do these toy models work?

**Fig. 2.**
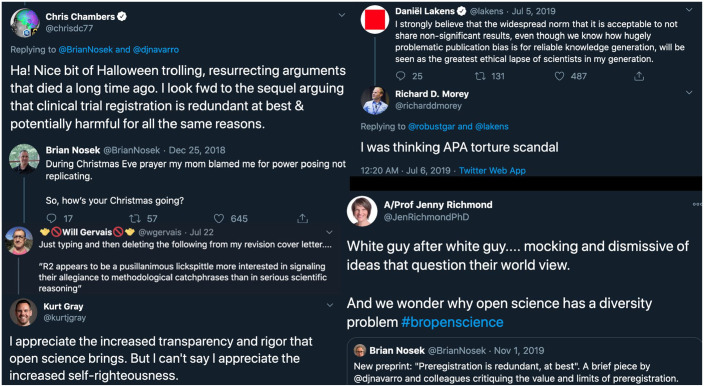
Potentially repellent messaging and incipient scientific polarization, broadcast via Twitter.

Many of us can easily call to mind examples of psychologists on one side of the reform aisle or another saying some objectionable things—methodological terrorists, shameless little bullies, holiday jokes about failed replications, needlessly pillorying Reviewer 2, arguing that the suppression of null results is worse than the suppression of human rights. And there are ample examples of social-media conflagrations over tone in communication and resultant polarization (Fig. 2). But is such tone actually harmful for achieving one’s desired ends?

Much ink has been spilled and many a verbal joust has been tilted over tone in scientific discourse. Ought we to moderate our tone? Is tone policing merely a cudgel wielded by elites against their uppity lessers? The rather tedious social-media conversations on this topic are sometimes called The Tone Debate. The goddamned tone debate. I hesitate to reopen this debate, but I hope it can be used to gently illustrate how modeling might in principle cut through verbal tedium and clarify things through making assumptions and trade-offs explicit.

This section develops a very simple model of the spread of open-science practices to ask—using simple, transparent, and, yes, unrealistic assumptions—whether tone could matter in the spread of open-science practices. Using verbal arguments, reformers have staked a pretty wide range of opinions regarding tone. I have argued that tone considerations could be viewed as necessarily tactical maneuvers used to reach and appeal to a broad and diverse audience (Gervais, 2017). Chambers countered that such care over inclusive tone is “a load of honking bullshit”^3^ (Chambers, 2017). Yarkoni argued that reformers faced a necessary trade-off between valuing inclusiveness/diversity and valuing rigor in metascientific reform (Yarkoni, 2019). These verbal arguments make little headway against each other. Might some rudimentary formal modeling help cut through this verbiage?

To develop a brief model, imagine a simple process by which people might acquire open science practices by simply observing others. To do so, an observer must, with some probability *P*(contact), encounter an individual who themself uses open-science practices. Once in contact with a demonstrator, the observer must, with conditional probability *P*(learn|contact), learn the practices from the demonstrator they’re in contact with. For the sake of simplicity, we’ll assume that nobody opts out of open-science practices once they are adopted. Under these sparse assumptions, a given observer adopts open-science practices based simply on the joint probability of contact with a demonstrator and learning from that demonstrator. Thus

$$P({\mathrm{adopt}}_{\mathrm{demo}})\;=\;P({\mathrm{contact}}_{\mathrm{demo}})\;P({\mathrm{learn}}_{\mathrm{demo}}|\mathrm{contact}).$$

**Fig. 3.**
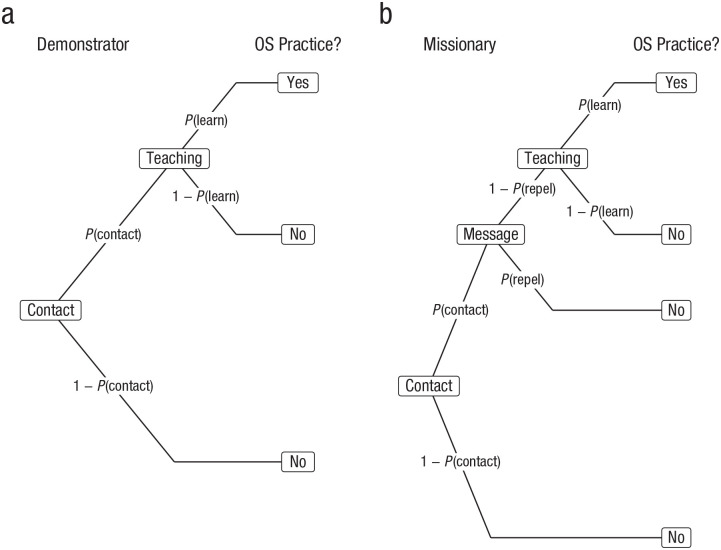
Multinomial processing tree illustrating conditional probabilities for the (a) demonstrator and (b) missionary strategies.

We can elaborate this model slightly to consider an alternative in which many people simply demonstrate their own open-science practices, as above and in Figure 3a. Meanwhile, others are active missionaries of the open-science gospel. They approach people and make a pitch, so to speak. However, the observer, with some probability *P*(repel|contact), finds the pitch repellent and opts out of the conversation—they do not even stick around to potentially pick up what the missionary preaches. Perhaps the missionary uses too many cat gifs, inappropriate jokes, or moralistic aggression. Assuming observers do not find the tone repellent, they proceed through potentially learning the open-science practices as before, at conditional probability *P*(learn|~repel), see Figure 3b. The probability of adopting open-science practices from a missionary is thus

$$\begin{array}{l} P({\mathrm{adopt}}_{\mathrm{missionary}})\;=\;P({\mathrm{contact}}_{\mathrm{missionary}})\;\\ (1\;-\;P(\mathrm{repel}\;|\;\mathrm{contact}))\;P({\mathrm{learn}}_{\mathrm{missionary}}\;|∼\;\mathrm{repel}). \end{array}$$

Figure 3 depicts the demonstrator and missionary strategies and their associated conditional probabilities to determine whether open-science practices are adopted by people in the context of each strategy.

We can then consider under which conditions the missionary strategy might outcompete the more basic process of simply observing people who demonstrate the practices. In this case,

$$P({\mathrm{adopt}}_{\mathrm{demo}})\;<\;P({\mathrm{adopt}}_{\mathrm{missionary}})$$

$$\begin{array}{l} P({\mathrm{contact}}_{\mathrm{demo}})P({\mathrm{learn}}_{\mathrm{demo}}|\;\mathrm{contact})\;<\;P({\mathrm{contact}}_{\mathrm{missionary}})\\ (1\;-\;P(\mathrm{repel}\;|\;\mathrm{contact}))P({\mathrm{learn}}_{\mathrm{missionary}}|\;∼\;\mathrm{repel}). \end{array}$$

If we assume that neither missionaries nor models differ in their contact rates, we can simplify the inequality to explore the impact of repellent messaging. Specifically, reducing reveals that the missionary strategy outcompetes the mere demonstrating strategy when

$$P(\mathrm{repel})\;<\;1\;-\;\frac{P({\mathrm{learn}}_{\mathrm{demo}})}{P({\mathrm{learn}}_{\mathrm{missionary}})}.$$

**Fig. 4.**
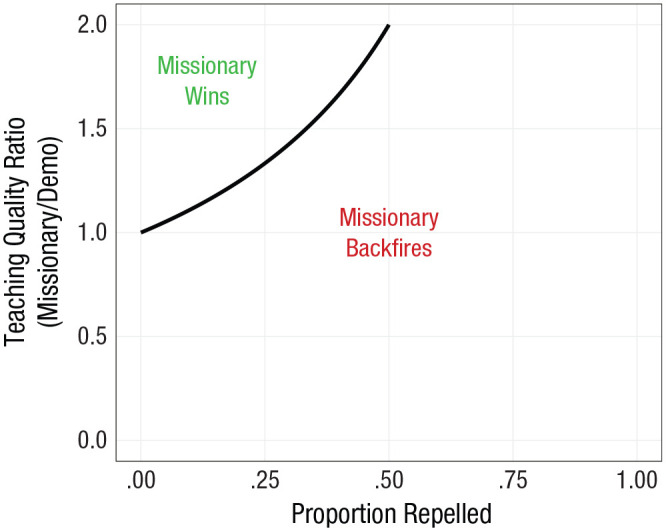
Modeling the tone debate: as the proportion of observers repelled by message tone (*x*-axis) increases, superior teaching (*y*-axis) is required.

In other words, in order for the missionary approach to prosper, people who encounter (and are not repelled by) missionaries must learn the resulting open-science practices at a higher probability than they would from mere demonstrators. In terms of teaching, the missionaries *must* offer a superior product. Further, the degree to which the teaching must be superior varies inversely with the proportion of people driven off by repellent messaging. This is not a linear relationship: If messaging alienates a fourth of the potential audience, a missionary must be a 33% better teacher, but if messaging alienates half of the audience, the teaching must be twice as effective. To make things quite concrete and clear, imagine that demonstrators successfully teach observers to adopt open-science practices half of the time. To compete, a missionary whose messaging alienates a quarter of observers would have to successfully convert two thirds of those they teach; a missionary who repels a third of observers would have to convert more than three quarters of those they teach; a missionary who alienates half would have to successfully teach every single individual who remained! Figure 4 plots the probability that a message is repellent against the ratio of teaching quality among missionaries and demonstrators.

Across a wide range of parameter values, missionaries are outcompeted by mere demonstrators. From this, a few observations can be made. First, missionaries *always* have to offer a superior teaching product—higher *P*(learn)—than demonstrators to offset those driven away by repellent messaging. The degree to which missionaries must be superior teachers increases steeply as their messaging becomes more repellent. Given this admittedly simplistic model, it looks like inclusive tone matters. If one’s goal is to increase those adopting open-science practices (assuming this dispersion follows something like the modeled process), it behooves reformers to seek out a wide audience with a more generally inclusive messaging. If one does use divisive and potentially repellent messaging, they ought to be especially mindful of this and realize that they will have to put in extra work elsewhere to compensate for those they have driven off through careless or intentionally abrasive tone. In practical terms, it is probably vastly easier to modulate one’s tone—reducing *P*(repel)—than to improve one’s pedagogy—increasing *P*(learn). One requires only self-control and effort, whereas the other requires learning new skills. Only by acknowledging the possibility that one’s tone might be overall counterproductive for a collective goal can individuals begin to grapple with the optimum ways to move forward in this example.

This is an overly simplistic toy model, and it makes some transparently silly assumptions. Some clear limitations are evident. For example, the model effectively assumed that demonstrators do not repel anyone (play with the formulae and you’ll see that *P*(adopt_demo_) is simply *P*(adopt_missionary_) with *P*(repel|contact) = 0. Surely no message is that innocuous! One could slightly elaborate the models to independently vary the rate of repellent messaging for different strategies. If one did this, one would conclude that the ratio of teaching effectiveness must outweigh the inverse ratio of repellent messaging: Bad tone must still be compensated for elsewhere in the learning pipeline. The model also ignored the possibility that some people might be actively attracted to certain forms—often aggressive forms—of tone. Indeed, the academic-Twitter slang “#bropenscience” refers to the sometimes cliquish trend of harsh and dismissive criticism in the name of open-science orthodoxy (Guest, 2019). This type of messaging might attract some like-minded folks and repel others. The model could be adjusted to add in this complexity. One potential outcome would be polarization within the community as groups cleave along the tone divide. Polarization like this is not a good sign for scientific progress (O’Connor & Weatherall, 2018).

This was just one silly toy example used to highlight how models—by making assumptions explicit and then quantifying their consequences—may help clarify thinking on methods reform and metascience. The next two sections quickly overview domains in which existing modeling efforts already generate insights that may be valuable to the methodological reform community, both in the domain of modeling scientific aspirations and in terms of modeling the processes of cultural change more broadly.

## Models of Scientific Aspiration

You have to decide what kind of difference you want to make.

— attributed to Jane Goodall

Should 100% of published studies be replicable? Is there an inherent tension between replicability and scientific discovery? If so, how should the two be balanced? What is the ultimate optimal outcome for scientific reform? What sorts of scientific communities are most conducive to truth-discovery?

Although answers to these questions are offered in print and via social media, the various answers are rarely explicitly theoretically grounded. Every few years there seems to be another spurt of pieces about rethinking our scientific discipline, reforming our incentives, or creating a new scientific utopia. And much of the advice and aspiration in these pieces is laudable and likely helpful! But much of it may prove to be more aspirational than realistic. Thankfully, much theoretical work exists that can help point out the types of science worth aspiring to.

### Replication versus discovery

Is there a tension between replication and discovery? What sorts of things ought a science to prioritize in order to maximize discovery of (in the words of Alexa Tullett, 2015) true things worth knowing? Does it make more sense to check via replication the current literature’s foundations? Forge forth with brand new investigations? Tweak theories?

To answer these questions, researchers could each go out and adopt different strategies, producing a blizzard of results of varying quality. We could wait some years, then produce metascientific assays of the resulting literature and make some pronouncements about which strategies yielded desired optima. Alternatively, we could try some theoretical modeling at the outset.

Devezer and colleagues (2019) present a thorough modeling framework for exploring these questions. This framework offers much to several ongoing discussions in the metascience community (the nature of replication, how to balance competing goals), and I hope it receives more widespread reading and discussion. They consider a scientific ecology in which different types of researchers focus on different aspects of the scientific process (replication, discovery, theory tweaking). They then consider how replication and discovery relate, how discoveries emerge, and how the ecosystem as a whole might be organized for optimal results.

At the level of individual energy, there is clear tension: A given replication project clogs up one’s resources that could have been put toward trying to discover something new, for example. Certain mathematical realities set upper bounds for replicability of observations in an uncertain world, and there are always trade-offs to be made when multiple scientific goods are desired. Although a given reported finding cannot in a sense be a discovery unless it is replicable, at the level of a scientific ecology, there is inevitable tension between discovery and replicability: There are possible scientific worlds in which everything is replicable and no discoveries are made (imagine a scientific ecology consisting solely of direct replications of the Stroop effect), as well as worlds in which many new discoveries are made while most apparent findings prove to be ephemeral (researchers prioritize ideas with low prior probability of truth). Rigorous theoretical modeling can help navigate this morass and point to potentially desirable optima, depending on participants’ own subjective weightings of various scientific utilities.

Combining simulation results, this modeling effort offers some intriguing insights. First, replication is obviously important, but is not the sole (or perhaps even most important) goal of science (Devezer et al., 2019). It is a necessary but not sufficient part of the scientific enterprise. Indeed, replication alone—divorced from theory—cannot even in principle halt the natural selection of bad science (Smaldino, 2019; Stewart & Plotkin, 2020). These models and others point out that individual effects can be highly replicable without being right (Baumgaertner et al., 2018; Devezer et al., 2019) and thus become incorrectly canonized.

One could maximize replicability—if that is one’s goal—simply by maximizing the prior probability that an effect is real: To maximize replicability, choose hypotheses one knows to be true! Of course by doing so, one largely gives up the possibility of genuinely new discovery. One could administer the Stroop task forever, rarely voyaging beyond the realm of certainty. Significant (replicable!) results would accumulate, to nobody’s excitement.

In contrast, one might prioritize discovery by choosing projects with low prior probabilities of success: Every study is a long shot, but every corroborated success is exciting! As a downside, however, a large number of the findings would simply be nonreplicable dreck. While some procedural steps such as increased sample size could firm up findings (Gervais et al., 2015), a risky strategy will inevitably produce more false starts (as well as discoveries!) along the way. Arguably, our field got in trouble by skimping on corroboration, but this does not diminish the potential of high-risk research; without it, we may evolve to prioritize slow, dull, conservative science (O’Connor, 2019a).

To optimize discovery in the face of potential false positives, some balancing is in order. Devezer and colleagues find that an ecology with a diversity of approaches—some replicators, some bent on discovery, some theoretical tweakers—outperforms others. Diversity of approaches and viewpoints is, per this model, integral to the success of the scientific ecology as a whole. This theme (diversity drives discovery) is apparent in a lot of other theoretical work on science, and we highlight converging sets of models that reach a similar conclusion about most fertile social ecologies for scientific progress.

### Optimal scientific ecologies

Theoretical work on replication and discovery highlights epistemic diversity as a key engine of scientific progress. This conclusion emerges again and again from quite different models of scientific networks.

Zollman (2007, 2010) modeled various network structures to evaluate the flow of information. Some networks were somewhat diffuse (linked by ties among adjacent individuals), whereas others were united by a central hub or were completely connected. A central hub could be thought of as a group of influential elites (for example they may organize conferences, popular symposia, or are otherwise “thought leaders” in the emerging group). Zollman also varied the strength of priors individuals would bear on a given problem. Somewhat paradoxically, networks with too much centrality in influential nodes or networks with too much interconnection tended to perform more poorly than those that contained looser agglomerations of subgroups. Taken broadly, this suggests that some transient diversity in views is a net benefit for the network as a whole. In contrast, too much influence from a central cadre can impede scientific progress.

Likewise, there are abundant examples of scientific communities converging on and lionizing false findings. As opposed to the corrosive influence of pathological or cynical corporate corruption, the ordinary workings of scientific networks may be one culprit (O’Connor & Weatherall, 2020). For example, scientists for decades largely overlooked work on the bacterial origins of ulcers simply because elites in their field had already converged on an alternative theory; there was insufficient attention paid to dissenting views. This type of scientific polarization (modeled and discussed well in O’Connor & Weatherall, 2018) is a constant threat in any scientific network in which elites wield undue levels of influence and are followed by a cliquish core group that views their own in-group science as epistemically superior to the critiques of outgroup members. Indeed, ignorance or denigration of work by what is seen as a rival camp is one big red flag for a polarized, and therefore probably suboptimal, science. It is a sign that cliques are potentially impeding progress.

Here it appears that a thriving and healthy science, per a fairly wide range of different models, emerges from promoting and cultivating diverse perspectives. In contrast, coalitionally polarized and overly conformist scientific ecologies tend to stifle progress. Regarding the threat of potential polarization, a key bellwether may be reception of dissenting ideas from individuals not seen as a central part of the core group. Are their points well considered? Is their work cited and discussed by group leaders? Or are their dissenting critiques silenced, ignored, ridiculed, or otherwise minimized? If people raise reasonable critiques of emerging movement orthodoxies (e.g., Szollosi et al., 2019), how is the critique received? The answer to these questions may forecast the strength of future science from that group. They are canaries in the coal mine of scientific polarization.

Beyond promotion of diversity and reduction of polarization, what other insights might models have for metascientific aspirations? Without dwelling too much on the details of any given modeling effort, some recurrent themes become apparent:

- current incentives lead to a cultural evolution of substandard science (Smaldino & McElreath, 2016);
- methodological change (e.g., badges for data sharing) without institutional change (e.g., funding and hiring incentives) are unlikely to fix this (Smaldino, Turner, & Kallens, 2019);
- those hiring norms can change via education (Gervais et al., 2015);
- reproducibility is neither necessary nor sufficient for scientific progress (Baumgaertner et al., 2018);
- scientific progress is facilitated by diverse viewpoints and hindered by cliquish devotion to emerging subcultures (Devezer et al., 2019; O’Connor & Weatherall, 2018; Zollman, 2010);
- if discovery is the primary aim of science, sometimes replicability must take a back seat (Devezer et al., 2019);
- replicability is no guarantee of truth (Devezer et al., 2019); and
- replication without theory cannot rescue us from bad science (Stewart & Plotkin, 2020).

None of these insights are trivial, many are counterintuitive, and they all offer suggestions for how to view scientific aspirations. We should pay attention to models, especially when they violate our intuitions or cherished notions. Models are stupid (Smaldino, 2017) and rely on deliberately unrealistic yet transparent (Guest & Martin, 2021) assumptions. Yet they can offer guidance about possible forces that could have created worlds like ours and highlight logical patterns that can shape our expectations about the scientific world we desire. At the very least, they are more transparently communicated than intuition-driven aspirations that may or may not *even in principle* generate the utopias they proclaim. Theory can help us calibrate our scientific expectations, if we’re willing to let our intuitions sometimes crash fatally against simple yet transparent assumptions.

This section outlined a few modeling results illustrating some insights about how an optimal science could look. Next we turn from these aspirational theories to theories about how change can actually occur. Clearly, a cultural shift is in order in science. How can we best shepherd this evolution? The next section illustrates results from basic models of cultural evolution, the scientific study of norms and institutional change over time, increasingly formalized and supported over the previous several decades.

## Models of Cultural Change

As humans, we have a mixed record with intentionally bringing about change. . . . I am convinced that evolutionary science provides an essential tool kit for making the world a better place.

— Atkins et al. (2019, p. 10)

**Fig. 5.**
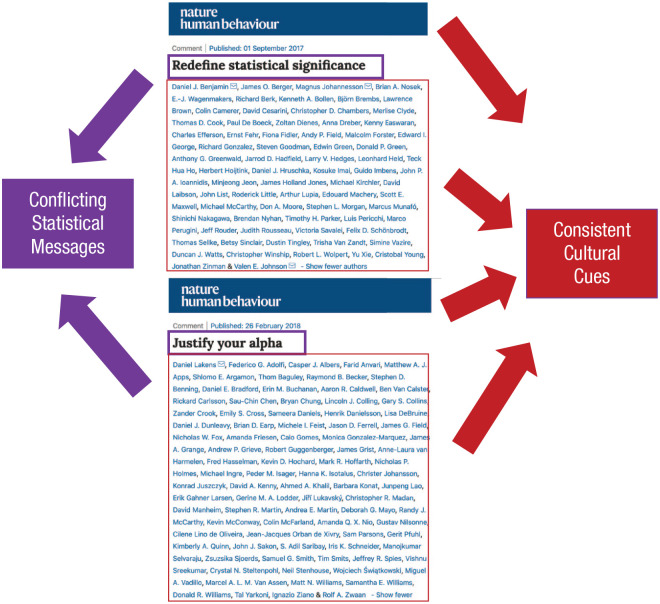
Mismatch between intended messages and cultural cues?

In the waning years of the first decade of the current millennium, a bitter war raged: Should the threshold of statistical significance, α, be redefined, abandoned, or arbitrarily set and then justified? The battle largely raged in the pages of *Nature Human Behaviour* (see Fig. 5), one of the more prestigious journals in the human sciences, as well as on the violent, blood-soaked e-steppes of academic Twitter. If a psychological scientist popped into existence, fully formed, capable of research, and armed with a basic statistical and methodological toolkit, they might be unsure of what statistical practices to adopt, given this exchange. Here are 174 eminent researchers, including some of the brightest lights in methods reform, unable to agree on concrete questions about statistical significance! What should a well-meaning scientist do?!

Our scientist may be confused about statistics from this exchange, but he or she will receive clear and consistent cues that one ought to publish in premier, high-prestige outlets such as *Nature Human Behaviour*. After all, that’s the behavior that nearly 200 scientific luminaries just so clearly demonstrated! Similar for other mega-author prestige and consensus papers in top academic journals nowadays: Whatever they argue, they also send clear cultural cues about other scientific values and publication practices, for better or worse. Figure 5 illustrates this potential mismatch between researchers’ stated objectives (statistical reform!) and the incidental cultural cues they simultaneously broadcast (publish prestigious or perish!).

Our hypothetical researcher is a naive cultural learner in this world and may rely on various cues to figure out how to succeed. A central thrust of this article is that those who wish to change scientific culture might benefit from stepping back and considering broader theory on how culture evolves in general. By this point, cultural evolution is a relatively mature (or at least maturing) discipline. Its seminal work is more than 35 years old (Boyd & Richerson, 1985) and has been continually refined and expanded to produce a thriving scientific subdiscipline that focuses intimately on questions regarding how norms, incentives, institutions, beliefs, and practices coevolve. It is well integrated within established theoretical traditions in evolutionary biology, backed by both formal models and empirical data, and it is well worth a perusal for anyone, such as science reformers, whose aim is explicitly about applied cultural evolution. What follows is merely an amuse-bouche, meant to whet appetites and stimulate interest in the theoretical foundations of cultural change.

### How cultures evolve

Methodological reform in science is fundamentally about culture change: How can we shift norms, incentives, and institutions to produce a more efficient and equitable scientific ecosystem? It is plausible that a theoretical understanding of culture change and norm evolution in general might prove helpful to those who wish to stabilize specific intended cultural changes (Bicchieri, 2016). Naturally, methodological reform goals may be met by various strategies, and many different theoretical perspectives can be harnessed in those efforts. People respond to incentives (economics), reinforcement (learning), and public shaming (reputation management and moral psychology). I encourage reformers to use all tools at their disposal, and I hope that a deeper appreciation for cultural evolutionary processes can add another theoretical arrow to the quiver. Theoretical knowledge of how cultures work may stimulate pragmatic approaches to cultural change in science.

Humans are not blank-slate cultural sponges. Instead, we appear to be equipped with specific mental adaptations (Barkow et al., 1992) including specific cognitive adaptations that enable the acquisition and transmission of cultural information (Rendell et al., 2011). Indeed, the human capacity for culture may explain our collective success as a species (Boyd et al., 2011; Mesoudi et al., 2006; Muthukrishna & Henrich, 2016).

Within the cultural evolutionary tradition, a few specific learning strategies might be of special relevance. *Conformist transmission* occurs when learners adopt strategies modeled by lots of others in their milieu (Henrich & Boyd, 1998). Crucially, it can lead to stable intergroup differences, as subpopulations converge on different norms. Beyond blindly following the majority, people can instead adopt various types of *success-biased transmission* strategies (Rendell et al., 2011), including *prestige-biased transmission* (Henrich & Gil-White, 2001), where learners pay deference for preferential access to elites within a group. This dynamic will no doubt be familiar to anyone who has attended an academic conference. Crucially, learners may not be able to directly ascertain what makes elites successful, and may overimitate them. This gives elites tremendous power to influence cultural transmission, whether they want it or not. Overimitation also means that features incidental to actual success will be copied, meaning that elites might unfortunately have to be very careful about what cues they project. Finally, learners must ensure that they are not being manipulated by Machiavellian or narcissistic elites: They must find ways to verify that elites actually hold the beliefs they espouse. Actions that would be costly to walk if elites did not believe their own talk—termed *credibility enhancing displays* or CREDs—are an often necessary assurance of sincerity among models (Henrich, 2009).

The combination of conformist transmission, prestige bias, and CREDs is a potent one, and it is easy to imagine how they could combine to reinforce or undermine suggested scientific reforms. Researchers who run larger, more labor-intensive studies take an inevitable hit to productivity (Bakker et al., 2012; Gervais et al., 2015). As a result, learners may infer that elites who do so are genuine in their beliefs that quality should trump quantity of publications. On the other hand, elites publishing opinion and recommendation pieces en masse may inadvertently be sending the signal, as mentioned previously, that success stems from frequent publication in high-status, high-impact journals—a message that may conflict with those elites’ stated opinions! Elites visibly encouraging constructive discourse online sends an active signal of inclusion; elites who either punch down or go quiet when flare-ups inevitably occur may inadvertently signal that abrasiveness and pugilism are part and parcel of metascience and reform, or at least tolerated.

Conformist transmission brings its own challenges and opportunities. The reformers are probably a numerical minority in psychological science. Public signals of practices such as preregistration thus can be risky. After all, one does not want to inadvertently signal that not adopting these practices is normative (Cialdini et al., 1990). As some reforms gain momentum, publicizing those relative gains may be more important than broadcasting absolute numbers. Another risk inherent to conformist learning is its ability to cleave and then stabilize groups (Henrich & Boyd, 1998). In-group labels, tags, and signals may help metascientists and reformers identify as a group, yet they can also make the group seem impenetrable to outsiders or foment destructive scientific polarization (O’Connor & Weatherall, 2018).

### Evolving better sciences

Some transient disagreement in beliefs is generally a good thing for a scientific community. Without diversity of belief, a community might fail to ever investigate a promising theory.

— O’Connor and Weatherall (2020, p. 48)

Science is a communal effort: We rely on each other for collaboration, critique, communication, and often consolation. The structures of our scientific networks and communities have emerged over decades of practice, pushed and pulled by various forces that may or may not be good for the grand enterprise of science. Many of the forces shaping scientific ecosystems have produced decidedly poor outcomes (Smaldino & McElreath, 2016). How might we intervene in this evolutionary process to produce scientific cultures that are more conducive to truth finding? How can we guide the evolution of our norms (Bicchieri, 2016)?

Cultural evolutionary work can suggest many features of successful cultures that are directly relevant to scientific cultures. In addition, modern philosophy of science is much more than Popper, Kuhn, and Lakatos. Philosophers of science are actively engaged in theoretical modeling of the processes active in the scientific enterprise, and tools such as cultural evolutionary models, network epistemology, and game theory illuminate many things that reformers should perhaps mind (Bicchieri, 2016; O’Connor, 2019a; O’Connor & Weatherall, 2018; Skyrms & Pemantle, 2009; Zollman, 2007). Integrating these perspectives, some tentative recommendations are possible.

Lone geniuses are overrated. Cultural success instead relies on collective efforts and pooled cognitive resources (Muthukrishna & Henrich, 2016). This means that scientific networks that are larger, less segregated, more diffuse, and more diverse will be more likely to converge on truth. This is a conclusion emerging from various independent lines of thought (Devezer et al., 2019; Muthukrishna & Henrich, 2016; O’Connor & Weatherall, 2018, 2020; Zollman, 2010). In an emerging community such as the methods reform/metascience community, this means that leaders should perhaps be mindful of opportunities for and challenges to growth, diffusion, recruitment, and diversification in views. The latter point—epistemic diversity—is especially important (Zollman, 2010) to avoid unnecessary polarization (O’Connor & Weatherall, 2018), which inhibits a search for truth (Devezer et al., 2019). This implies an active openness to even opinions challenging emerging reform orthodoxies (Navarro, 2019; Szollosi et al., 2019; van Rooij, 2019). The alternative is a subcommunity of scientists who preferentially trust science from in-group members and ignore or dismiss the work of outsiders, leading to entrenched false beliefs and difficult-to-shake myopias (O’Connor & Weatherall, 2020). This polarization needlessly puts blinders on the scientific process, as in-group loyalty trumps openness to divergent and potentially important lines of thought (O’Connor & Weatherall, 2018).

Beyond openness to divergent opinions on emerging topics, an emphasis on diversity, depolarization, and growth implies that a frequent audience for metascientific and reform messaging is not other reformers, or even advocates of the status quo, but rather the vast middle that is likely uncertain on many methodological issues and using perhaps incidental cultural evolutionary cues to determine their responses. Harsh, abrasive scientific criticism and mockery of substandard articles or even widespread mockery of entire domains of research may appeal to some, but it may also make an untactical appeal to observers (Gervais, 2017; Navarro, 2019). Thus, consistent with our pet model, concern for inclusive messaging on behalf of both reformers and status-quo-ers, far from being “a load of honking bullshit” (Chambers, 2017), is actually a strongly theoretically supported recommendation for generating the type of diverse, nonpolarized, broad scientific community that has a chance to actually solve the tough cultural evolutionary challenges we currently face (Muthukrishna & Henrich, 2016; O’Connor & Weatherall, 2018; Zollman, 2010). We ignore this theoretical insight—independently derived numerous times and corroborated across disciplines—at our own peril.

### Summary

This is far from a full treatment of cultural evolution and its associated developments from biology, anthropology, psychology, and philosophy of science. Accessible treatments are widely available (Bicchieri, 2016; Mesoudi et al., 2006; Richerson & Boyd, 2008). Instead, I wanted to use basic concepts from cultural evolution to illustrate ways in which reformers might be especially mindful of the cultural signals various choices send and the likely outcomes that result from them.

When facing entrenched maladaptive scientific norms that place new methods at a competitive disadvantage (Smaldino & McElreath, 2016), reform faces an uphill battle. Practical reform needs every tool at its disposal, including tools developed and refined in other disciplines to answer quite different questions about how to change norms (Bicchieri, 2016). In order to grow a collective brain capable of improving science (Muthukrishna & Henrich, 2016), our best theory suggests that larger, more inclusive, more diverse, more integrated networks are in order (e.g., Devezer et al., 2019; O’Connor & Weatherall, 2018, 2020; Zollman, 2007, 2010). Everyone’s behavior—thanks to conformist transmission and CREDs—can serve as a catalyst, although prestige biases make elites especially important. We are all sending cues to each other, and our scientific culture will evolve according to the cues we collectively send and attend to, for better or worse.

## From the Natural Selection of Bad Science to the Intelligent Design of Better Science

We’re all making it up as we go along, to the best of our ability, hoping not to make a mess of everything. Under the circumstances, I think a little modesty in our scientific and statistical claims would be in order, no?

— Danielle Navarro (2019, p. 11)

Our current scientific ecosystem is unhealthy. Cheap, low-effort, unreliable science can spread at the expense of slower, more reliable work (Bakker et al., 2012; Gervais et al., 2015; Smaldino & McElreath, 2016). Fortunately, we have the opportunity to clean up the mess we’ve evolved.

We are an evolved species, genetically and culturally. But we also have the intelligence to guide the evolution of our cultures. An understanding of evolutionary forces can shed light on how societal ills are maintained, inside (Smaldino & McElreath, 2016) and outside (O’Connor, 2019b) of science. But knowledge is power! Once we understand the forces that created problems, solutions might be more possible. Guided cultural evolution can be practiced at various levels of social organization (Atkins et al., 2019; Bicchieri, 2016; Wilson, 2011) and is well worth attempting in science (O’Connor, 2019a; Smaldino, 2019; Stewart & Plotkin, 2020).

A vibrant methods reform and metascience community has sprung up in psychology. We seek to reshape the scientific ecology that we have (likely unwittingly) allowed to evolve, an ecosystem in which the factors driving individual success erode the collective enterprise of science. Our metascience and reform movement is characterized by lofty goals and a tireless passion for science. I argue that it can maximize its odds of success by drawing on all available theoretical tools, especially turning to tools that have originated and fermented in areas adjacent to psychology, including philosophy of science and cultural evolution.

Given the cultural evolutionary forces that drive the spread of substandard science (Smaldino & McElreath, 2016), it only makes sense to turn to core evolutionary principles to turn the tide and intentionally evolve or design a better scientific ecosystem. This endeavor, ultimately, is a project of guided cultural evolution (Atkins et al., 2019; Bicchieri, 2016; Wilson, 2011). So let us turn to the best available theories to sharpen our metascientific projects, tune our scientific aspirations, and change the norms and institutions we have inherited.

Theory gives us a clue how to proceed. Theory can spur the evolution of better science in domains in which technical, methodological, and statistical tweaks will likely prove insufficient (O’Connor & Weatherall, 2020; Smaldino, 2019; Stewart & Plotkin, 2020; Szollosi et al., 2019; van Rooij, 2019). Theory can help us choose and interpret replication projects (Field et al., 2019). It can help us hone our statistical intuitions about what replication rates are or ought to be. Theory can help us set goals for reform of the field to maximize the scientific desiderata we most value (Devezer et al., 2019). It can make our forensic assays of the field more efficient and meaningful (Field et al., 2019). Theory can even give us hints as to what cues we may (even inadvertently) be sending observers, perhaps undermining the types of communities most likely to actually solve the practical challenges science faces today (O’Connor, 2019a; O’Connor & Weatherall, 2018; Zollman, 2010).

We have passively evolved a toxic scientific ecosystem. Perhaps by embracing relevant theory, including work from outside psychology, we can intelligently design a healthier one for future generations of scientists.

## Transparency

*Action Editors:* Travis Proulx and Richard Morey

*Advisory Editor:* Richard Lucas

*Editor:* Laura A. King

## Notes

1. I thank Reviewer 2.

2. For example, Jones tweeted “The myth that psychological findings in MTurk or WEIRD samples are ‘unrepresentative’ of humans needs to go away. This line of reasoning assumes a priori that there is a strong moderation of psychological effects by demographic characteristics” (2019b) and then tweeted “As it turns out, such moderation is extremely rare. See for instance the results from Many Labs 2” (2019a).

3. The full quote was as follows: “There’s this idea that open science will attract more ‘disciples’ if it comes across as having a more positive, inclusive tone. Goodness me, what a load of honking bullshit this is.”
